# Transforming Perioperative Care: Evolving Paradigms of the Expanding Role of Regional Anesthesia and Acute Pain Management

**DOI:** 10.3390/jcm14176257

**Published:** 2025-09-04

**Authors:** Eleni Moka

**Affiliations:** Faculty of Medicine, School of Health Sciences, Aristotle University of Thessaloniki, 54124 Thessaloniki, Greece; mokaeleni@hotmail.com

Over the past few decades, the field of regional anesthesia (RA) has witnessed a period of profound advances, extraordinary progress, and dynamic transformation. Once regarded as a niche domain within anesthesiology—primarily confined to selected surgical specialties—RA has evolved and has now emerged as a foundational pillar of modern anesthetic practice and a key enabler of individualized perioperative care. This evolution has been fueled by a confluence of scientific breakthroughs, clinical innovations, and technological advancements, all contributing to reshaping RA to be a refined, sophisticated, evidence–based, and widely implemented approach in contemporary medical practice. Its seamless integration into acute pain management protocols and perioperative clinical pathways has far-reaching implications on patient care, with recognized benefits that extend beyond superior analgesia to include enhanced recovery, greater patient satisfaction, reduced opioid dependence via opioid-sparing strategies, cost efficiency, and improved overall system performance. Ultrasound-guided RA (UGRA) techniques have revolutionized RA delivery, establishing a new “gold” standard of care thorough increased precision, safety, and ease of performance. Consequently, the accessibility, global clinical adoption, and popularity of RA have expanded dramatically. In the current era of patient-centered care, RA and acute pain management are no longer considered adjunctive tools but are viewed as integral components of a comprehensive, holistic, personalized perioperative strategy [1,2,3].

This progression has been propelled by the convergence of an expanding body of rigorous, high–quality clinical research, refined anatomical insights and complementary understanding, advanced pharmacologic options, and a shift towards value-based, outcome-driven healthcare. By minimizing opioid exposure and its associated risks while facilitating earlier mobilization and smoother recovery trajectories, the synergistic application of RA and acute pain management protocols exemplifies the principles of personalized and precision medicine. Yet, despite these strides, the field has not yet reached its full potential and continues to evolve. Emerging peripheral nerve blocks (PNBs), new-generation pharmacological adjuvants, and updated clinical protocols and practices still present opportunities for further refinement, validation, widespread dissemination, and broader implementation. The continued pursuit of innovation, safety, and patient-centered excellence will be essential to realizing the full potential of RA and acute pain management in the years ahead [2,3,4].

This Special Issue of the *Journal of Clinical Medicine* titled “Advances in Regional Anesthesia and Acute Pain Management” presents a timely, comprehensive, and multidimensional exploration and overview of this rapidly advancing discipline. Comprising eighteen rigorously peer-reviewed articles, authored by leading international experts, this collection offers an in-depth examination of the latest advances in RA and acute perioperative pain management. The contributions span a broad range of critical topics, including detailed anatomical considerations, technical innovations, pharmacologic developments, clinical applications and outcomes, safety protocols, and educational methodologies. Collectively, these studies not only synthesize current best practices but also illuminate ongoing challenges, emerging questions, and key opportunities that will shape the next phase of clinical evolution. At the core of this Special Issue lies the shared conviction that RA is uniquely positioned to advance the goals and support the evolving paradigm of modern perioperative medicine—delivering care that is not only effective and efficient but also individualized, humane, and grounded in the principles of precision and compassion. Unlike systemic analgesics, which affect the entire body and carry a higher burden of adverse effects, RA techniques enable site-specific interventions that can be adjusted to suit individual anatomy, the type of surgical procedure, patient comorbidities and individual characteristics, and recovery goals. These tailored strategies improve not only the physiological outcomes—such as pain control, reduction in postoperative nausea and vomiting, and early mobilization—but also psychological well-being and overall satisfaction. Importantly, in an era of escalating healthcare costs, RA contributes to value-based care through decreased hospital stays, reduced reliance on opioids, and improved long-term functional recovery.

The principle of a personalized approach to perioperative care is vividly exemplified in the population-based study by Cozowicz C. et al., which serves as a featured contribution within this Special Issue [5]. In this large-scale analysis encompassing data from over 1.3 million patients undergoing hysterectomy, the authors investigated outcomes associated with multimodal analgesia strategies, including RA implementation. Their findings revealed substantial improvements in postoperative outcomes, with multimodal analgesia being associated with a 35% reduction in overall complications, a 24% decrease in opioid consumption, and a 14% shorter hospital length-of-stay. A closer examination showed that respiratory complications were reduced by approximately 40%, cardiac complications decreased by 30%, and genitourinary complications declined by 40% when compared to patients who did not receive multimodal analgesia. Furthermore, the study demonstrated a stepwise reduction in opioid prescriptions, with the incremental application of additional analgesic modalities. The large-scale, high-volume, and real-world nature of this study offers compelling and generalizable evidence in support of multimodal pain management strategies. These findings make a persuasive case not only for the routine implementation of such protocols in gynecologic surgery but also for their broader adoption as a model of best practice across various surgical specialties. At a time when healthcare systems are striving to optimize outcomes within constrained resource environments, the implications of this research are both timely and highly consequential.

In orthopedic surgery—a field where postoperative pain is often profound and functional recovery depends on early mobilization—RA has consistently demonstrated substantial benefits. Multiple studies in this Special Issue reinforce the clinical value of PNBs in enhancing analgesia and reducing reliance on systemic opioids. Beric S. et al. provided clear evidence that supplementing spinal anesthesia with adductor canal and sciatic nerve blocks in patients undergoing anterior cruciate ligament reconstruction significantly improved immediate postoperative pain relief, preserved range of motion, and led to a marked reduction in opioid consumption [6]. As such, their study underscores the utility of targeted, site- and procedure-specific nerve blocks in optimizing recovery trajectories in procedures characterized by a high nociceptive burden. Complementing these findings, Viderman D. et al. conducted a comprehensive systematic review and meta-analysis, examining the use of gabapentinoids in joint arthroplasties [7]. While their analysis of over 1200 patients demonstrated a consistent, albeit modest, opioid-sparing effect, the overall benefit–risk profile of gabapentinoids remains a topic of ongoing debate and, according to the authors, pain reduction was not clinically relevant. Sedation was not evaluated in their study and, if taken into account, it might have influenced their conclusions. An important limitation of this study was that different gabapentinoids, their administration times, and dosages, as well as varying intraoperative management protocols, were pooled together. These agents have been incorporated into many multimodal analgesic regimens, based on early enthusiasm regarding their potential to reduce central sensitization and enhance postoperative pain control [8]. Nevertheless, more recent evaluations have highlighted concerns about their limited efficacy in reducing pain scores in the immediate and longer postoperative period and their association with side effects such as sedation, dizziness, and impaired balance—factors that could hinder early mobilization and increase the risk of falls in the orthopedic population [9,10]. As such, the role of gabapentinoids in orthopedic pain management is increasingly viewed as context-dependent, requiring careful patient selection, as well as dose titration and meticulous adjustment. Their inclusion in perioperative analgesia protocols should be informed by a nuanced understanding of both their pharmacodynamics and the evolving evidence base [10]. The juxtaposition of studies on RA techniques and gabapentinoids highlights the complexity of contemporary perioperative pain management, where no single approach or intervention suffices or can address the diverse needs of every patient. Instead, these findings reinforce the importance of individualized, evidence–based strategies that integrate pharmacologic, procedural, and functional considerations to achieve optimal outcomes while minimizing adverse effects.

Among the most clinically relevant and increasingly recognized challenges addressed in this Special Issue is the phenomenon of rebound pain (RP). Defined as a marked resurgence of intense pain following PNB resolution, RP has the potential to significantly disrupt the postoperative recovery process. It can impair patient satisfaction, delay functional rehabilitation, and in some cases, it may lead to increased reliance on rescue opioid analgesics [11]. Lee B. et al. provided valuable insights into this issue by directly comparing the efficacy of continuous versus single-shot superior trunk blocks in patients undergoing arthroscopic shoulder surgery [12]. Even when the analgesic efficacy of single-shot blocks was prolonged with intravenous dexmedetomidine as an adjuvant, their results demonstrated that continuous nerve blocks provided smoother, more stable, and more reliable postoperative analgesia while significantly reducing both the intensity and the frequency of RP following arthroscopic rotator cuff repair. These findings align with the growing clinical consensus that RP is a predictable phenomenon, and the expert opinion is that it must be anticipated and proactively managed, particularly in outpatient and ambulatory surgical settings, where patients are often discharged before PNBs wear off completely [13].

Indeed, the incidence of RP is notably higher in ambulatory patients and appears to be modulated and influenced by a combination of patient-specific, surgical, and anesthetic factors. Strategies to mitigate RP include thorough preoperative patient education, the implementation of multimodal analgesic protocols, the use of continuous PNB techniques, and the administration of systemic agents, such as intravenous dexamethasone. While RP may not universally lead to increased overall opioid consumption nor uniformly affect functional recovery or patient satisfaction scores, it is nonetheless a source of significant discomfort for surgical patients. Interestingly, despite experiencing RP, the majority of affected patients report that they would still opt for PNBs in future operative procedures, underscoring the perceived value and benefits of RA techniques. Moving forward, further research should aim to establish predictive models and tools to identify patients at higher risk for developing RP and to explore novel pharmacological or procedural modifications and strategies that may help reduce RP incidence and improve the continuity of postoperative analgesia [11,13,14].

The pharmacological enhancement of the RA technique efficacy represents another area of active investigation. Studies in this Special Issue explore the use of adjuvants to extend fascial plane block duration, improve postoperative analgesia quality, and mitigate side effects. Korkutata Z. et al. compared dexmedetomidine and tramadol as adjuvants to bupivacaine in transversus abdominis plane (TAP) blocks, finding dexmedetomidine to be superior in both analgesic duration and hemodynamic stability [15]. Urfalı S. et al. examined the impact of dexamethasone and dexmedetomidine on bupivacaine TAP blocks in cesarean section patients, observing longer-lasting analgesia and a reduced need for rescue medication [16]. Dexamethasone, due to its delayed onset but extended duration, was found to achieve lower pain scores and higher patient satisfaction scores. These findings are important not only for enhancing the RA efficacy but also for reducing the systemic opioid burden, a key public health priority in the context of the opioid epidemic.

In addition, fascial plane blocks—one of the fastest-growing areas in RA—are also well represented. M. et al. conducted a head-to-head comparison of the serratus anterior, erector spinae, and paravertebral blocks for breast cancer surgery [17]. Their results demonstrated comparable intraoperative analgesic efficacy across all three RA techniques, but with the serratus anterior block emerging as equally effective as the paravertebral block for reducing postoperative pain; it was determined to be the most practical option due to its ease of application and lower complication risks. Kim S. et al. extended the utility of the erector spinae plane block to cardiac surgery, showing its effectiveness, as a supplementary approach to cardiac anesthesia, in reducing opioid use and ICU length-of-stay in off-pump CABG patients [18]. These studies illustrate the versatility of fascial plane blocks and highlight their value in patients who are not suitable candidates for neuraxial techniques due to anticoagulation or hemodynamic instability.

Furthermore, the anatomical basis of PNBs is explored in detail in several contributions in this Special Issue. Bjorn S. et al. provided an important update on the medial femoral cutaneous nerve, demonstrating its anatomical consistency and clinical relevance in total knee arthroplasty (TKA) [19]. Inadequate coverage of this nerve may contribute to residual anteromedial knee pain postoperatively—a persistent issue in a subset of patients, although further trials are mandated to investigate whether its blockade translates into a clinical effect on postoperative pain after TKA or if it can be used for diagnosis and interventional pain management for chronic neuropathic pain due to its damage during surgery. Moreover, Staikou C. et al. examined the role of pericapsular nerve group (PENG) block in major hip surgery, concluding that it offers better postoperative analgesia with possibly less opioid consumption, prolonging the time to the first analgesic without the significant common side effects of anesthesia/analgesia or duration of hospital stay [20]. Similarly, Muse I. et al. reviewed the PENG block and its relevance to hip analgesia and highlighted a new block that anesthetizes nerves to the posterior capsule, superior gluteal nerve, and the nerve to the quadratus femoris muscle, called the posterior pericapsular deep-gluteal block, suggesting that complete analgesia for hip fracture surgery is possible, thus optimizing perioperative pain management [21]. These studies underscore the continued importance of anatomical precision in the development and refinement of RA techniques. As new blocks emerge and old ones are modified, anatomical validation remains essential to ensuring efficacy and avoiding inadvertent injury during PNB placement or surgery.

Notably, RA is no longer confined to adult surgical populations. Capuano P. et al. presented a compelling case study involving a pediatric renal transplant recipient, managed with a continuous erector spinae plane block [22]. The child experienced excellent pain relief, avoided systemic opioids, and had an uneventful recovery. This case, while limited in generalizability, offers a valuable template for expanding RA into pediatric and transplant populations—areas that have historically been underrepresented in RA clinical research. With proper training, institutional support, and vigilant monitoring, RA techniques can be safely employed in even the most vulnerable patient groups.

Education and training are also central to safe, consistent, and effective RA application. Seybold B. et al. explored the learnability of an ultrasound-guided cervical plexus blockade, used for carotid endarterectomy, and it demonstrated high success rates among anesthesia trainees, following brief yet structured instructional sessions [23]. Their findings highlight the rapid acquisition of this skill, a notably low failure rate, and a reduction in complications, particularly among anesthesiologists already familiar with other UGRA techniques. Their study affirms the critical role of simulation-based learning, hands-on mentorship, and stepwise skill-building in mastering anatomically complex and technically demanding PNBs [24,25]. As RA continues to expand in clinical scope and complexity, the standardization of educational frameworks will become increasingly important [26]. Incorporating simulation platforms [27], artificial intelligence (AI)-driven feedback systems [28], structured performance checklists, and objective competency assessments into training curricula will be essential for ensuring proficiency, quality, and patient safety. Furthermore, as RA becomes more advanced and widely accessible, remote learning solutions and virtual education models offer promising strategies to overcome geographical and institutional barriers to training [29]. These innovations may significantly expand global access to high-quality RA education, ultimately contributing to better outcomes and safer care across diverse practice settings.

Safety is a recurring theme in this Special Issue, particularly in relation to complex or controversial areas of practice. Hilber N. et al. addressed the concern that RA may mask symptoms of acute compartment syndrome in limb trauma surgery [30]. By reviewing 35 case reports and series, they concluded that RA techniques—especially when low-concentration local anesthetics are used—do not prevent timely diagnosis, especially when combined with vigilant clinical monitoring. Their analysis highlights the importance of interdisciplinary collaboration, patient education, and protocol-driven practice in high-risk settings. Safety is not a passive outcome but an active process requiring commitment, communication, and continual reassessment.

The international scope of this Special Issue deserves special mention. Authors from Europe, North America, Asia, and other regions bring diverse perspectives, practices, and innovations to the table. This geographic diversity underscores both the universal applicability of RA and the importance of context in its implementation. In low- and middle-income countries, for instance, barriers such as limited ultrasound machines access, staffing shortages, and insufficient training infrastructure must be addressed creatively. Solutions such as mobile simulation labs, remote mentoring platforms, and scalable educational programs may offer a roadmap for global equity in RA provision to our patients [31].

Technology is playing an increasingly important role in the evolution of RA. Emerging tools such as AI-assisted image interpretation, augmented reality-guided needle placement, and automated injection systems promise to enhance block accuracy, reduce operator variability, and improve patient safety. As these technologies mature, their integration into clinical practice must be guided by rigorous validation studies and cost-effectiveness analyses [32]. Moreover, the integration of RA data into electronic health records and clinical dashboards will enable real-time quality monitoring and performance improvement, ensuring that best practices are not only defined but also consistently implemented.

Looking forward, several strategic priorities emerge for this specialty. First, continued investment in high-quality research, including multicenter randomized trials and longitudinal outcome studies, is essential to answer remaining questions about block efficacy, safety, and comparative value [33,34]. Second, more attention must be paid to long-term outcomes, such as chronic postsurgical pain, return to function, and health-related quality of life [1,35]. Third, health economic studies should quantify the cost–benefit profile of RA techniques across different healthcare systems and patient populations. Fourth, efforts to standardize documentation, procedural nomenclature, and outcome reporting will enhance the comparability and generalizability of future research [1,35,36].

In conclusion, the articles presented in this Special Issue reflect the strength, depth, and maturity of RA as a discipline. They showcase a field that remains innovative, is evidence-based, and globally engaged; it is a field committed to improving patient care through precision, empathy, and scientific rigor. The knowledge shared herein not only advances the science of RA but also reaffirms its role as a cornerstone of modern perioperative medicine. The editorial team extends their sincere appreciation to all authors, peer reviewers, and collaborators who contributed to this issue. Their dedication, vision, and scholarly excellence have made this publication a valuable resource for clinicians, educators, and researchers alike. It is hoped that the insights gathered here will inspire continued inquiry, collaboration, and innovation in the service of better, safer, precise, and more personalized perioperative pain management worldwide.
